# In-Vehicle Situation Monitoring for Potential Threats Detection Based on Smartphone Sensors

**DOI:** 10.3390/s20185049

**Published:** 2020-09-05

**Authors:** Alexey Kashevnik, Andrew Ponomarev, Nikolay Shilov, Andrey Chechulin

**Affiliations:** St. Petersburg Federal Research Center, Russian Academy of Sciences (SPC RAS), St. Petersburg Institute for Informatics and Automation of the Russian Academy of Sciences, St. Petersburg 199178, Russia; ponomarev@iias.spb.su (A.P.); nick@iias.spb.su (N.S.); chechulin@comsec.spb.ru (A.C.)

**Keywords:** intelligent transportation systems, threats classification, vulnerabilities detection, smartphone sensors

## Abstract

This paper presents an analysis of modern research related to potential threats in a vehicle cabin, which is based on situation monitoring during vehicle control and the interaction of the driver with intelligent transportation systems (ITS). In the modern world, such systems enable the detection of potentially dangerous situations on the road, reducing accident probability. However, at the same time, such systems increase vulnerabilities in vehicles and can be sources of different threats. In this paper, we consider the primary information flows between the driver, vehicle, and infrastructure in modern ITS, and identify possible threats related to these entities. We define threat classes related to vehicle control and discuss which of them can be detected by smartphone sensors. We present a case study that supports our findings and shows the main use cases for threat identification using smartphone sensors: Drowsiness, distraction, unfastened belt, eating, drinking, and smartphone use.

## 1. Introduction

Nowadays, intelligent transportation systems (ITS) are attracting more and more research and development activities. There are a lot of conferences (IEEE International Conference on Intelligent Transportation Systems, IEEE Intelligent Vehicles Symposium postponed, Vehicle Technology and Intelligent Transport Systems, and etc.) and journals (IEEE Transactions on Intelligent Transportation Systems, IEEE Transactions on Intelligent Vehicles, and etc.) that join together researchers in the area. In addition, car manufactures are widely implementing ITS technologies in the produced vehicles. Such technologies include advanced driver assistance systems (ADAS) [1], vehicle-to-vehicle (V2V) Communication [2], driver monitoring systems [3]. The main task of ITS is to solve large-scale challenges of transportation infrastructure (i.e., routing, congestion prevention, and safety). Modern sensors for data collection and artificial intelligence solutions for its processing are widely used in ITSs. Information from sensors is joined into a holistic representation of the traffic situation and employs various predictive models to estimate future situation.

Driver Assistance Systems (DAS) support vehicle driver with the following features: antilock-breaking, adaptive cruise control, parking or lane change assistance, drowsiness monitoring, and etc. The main difference between DAS and ITS is that DAS provide functions to support a particular driver, while ITS are aiming at more global tasks related to traffic organization in a particular region.

The basic entities considered in the paper are the driver, environment (that includes vehicle the driver controls, road situation, and weather), and DAS. The road situation includes an infrastructure and other vehicles. The infrastructure includes information signs, toll points, monitoring, and parking systems. DAS are designed to support the driver in vehicle cabin and reduce accident probability, they reduce the level of both threats that the driver can bring to the environment, and threats that the environment can bring to the driver. However, DAS can also bring some threats for both the driver and the infrastructure.

Modern DAS are cyber-physical systems, software and hardware components, which can cause potential threats for them. At the moment, before automated vehicles are widely used, the human driver is the main part of any transportation system. When the driver is added to such cyber-physical system it becomes a socio-cyber-physical system with more components and threats. As a result, potential threats have to be considered taking into account interaction of DAS with the human. The paper makes a first step in this direction. Just as DAS, Driver Monitoring Systems (DMS) can both reduce some threats under the human machine interaction, and cause new threats during the interaction.

The main contribution of the paper is to identify possible threats in vehicle cabin, classify them, and discuss which of them can be detected by smartphone sensors (including front and back cameras, microphone, positioning system (GPS/GLONASS), accelerometer, magnetometer, and gyroscope). We believe that the analysis presented in the paper is important in three ways. First, it encompasses further research on in-vehicle monitoring, especially the line of research focused on using minimal (most accessible) hardware for increasing driver’s safety. Second, it provides a functional framework for in-vehicle monitoring applications. Finally, it allows one to systematically evaluate in-vehicle driver monitoring applications, in particular, the completeness of threats coverage.

In the paper we use the following standard terminology from the IT security domain [4]. Vulnerabilities are weaknesses that might be used to bring undesired damage. Threats are circumstances that potentially can cause undesired damage. Attacks are actions aimed at “the exploitation of vulnerabilities by threats”.

The rest of the paper is organized as follows. Related work in the topic of ITS, information flows in such systems as well as human-computer threats detection is presented in Section 2. Results got on the basis of the system analysis are presented in the Section 3. Research questions are discussed in Section 4. Main results are summarized in Section 5.

## 2. Related Work

Most of the research related to in-vehicle threat detection considers vehicles, and all its information systems, as a cyber-physical system. Therefore, from this point of view, a threat (or attack) detection is considered in the realm of cyber-security. However, the human driver and his/her interactions with the transportation system participants and various smart tools are inextricable part of this socio-cyber-physical system. In this regard, we argue that the scope of safety and security assurance should be extended. In this section, we enhance our previous paper [5] and discuss related work in the both directions, preparing background for the synthesis.

### 2.1. Cyber-Physical Centered Point of View

The cyber-security research in transportation systems has three major goals: (1) to identify and to classify the existing threats and vulnerabilities immanent to cyber-physical transportation systems, (2) to develop risk assessment methodologies, and (3) to propose new technological and engineering countermeasures to the existing threats. Cyber-security in transportation systems is actively explored in at least two levels of granularity: Intra-vehicle level (e.g., in-vehicle communication between components via controller area network (CAN) buses [6]), and inter-vehicle level (e.g., communications between various components of a large-scale intelligent transportation system [7,8]).

A very productive approach to threats analysis is to consider the system as a whole, identify possible parts of it and then discover security problems that might be associated with each part of the system. This is done, for example, in the paper [9], exploring the vulnerabilities of connected and autonomous vehicles (CAV) and proposing a reference CAV architecture tailored for attack surface analysis.

Standardized guidelines also address the problem of cyber-security of connected vehicles, providing a number of methodologies for evaluating vehicle design decisions (with SAE J3061 being a notable example). These guidelines are trying to account for both security and safety concerns both of which are crucial for transportation systems. It means that the so-called HARA (hazard analysis and risk assessment) and TARA (threat analysis and risk assessment) has to be done jointly and may possibly intertwined [10,11].

As sensors play a very important role in CAVs, the CAVs’ cyber-security is also connected with the area of sensor security, which is also a developed area with several existing security assurance methodologies and practical solutions (e.g., ISO/IEC 15408 Common Criteria to solve specific security problems of sensors [12]).

Some works classify threats from different points of views. For example, Parkinson et al. [13] classifies cyber CAVs vulnerabilities based on the CAV automation levels of and knowledge gaps existing in the security of various cyber CAV components. Cyber security issues are analyzed in [14] but in a more narrow domain of Vehicle Ad Hoc Networks (VANETs), including vehicle-to-vehicle and vehicle-to-roadside communications. The classification presented in [15] is based on an analysis of existing security standards and threat countermeasures.

Possible cyber, physical and cyber-physical attack classification is presented in [16] together with a taxonomy of intrusion detection software (IDS). Three main cyber-physical attack targets are identified: Confidentiality, integrity, and availability. However, this work mainly looks at IDS classification rather than the threats themselves.

The authors of [17] classify the environment-vehicle-driver threats for cyber, physical and cyber-physical. They also represent a two-dimensional classification, where the other dimension is constituted of vulnerabilities, attacks and threats.

### 2.2. Human-Centered Point of View

**Figure 1:**
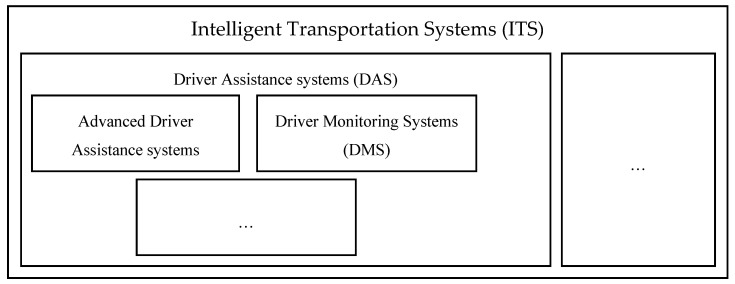
ITS Classification for vulnerability detection in vehicle cabin.

We consider two roles of human in modern ITS. Human is the user for ITS, he/she gets benefits from the provided services, and human as information source [18]. Based on the considered roles, we identify human as a threat object and human a threat source. We consider these roles in detail in the section. Figure 1 shows relationships between ITS, DAS, ADAS, and DMS considered in the paper. Since we consider only in-cabin vulnerability detection, we concentrate on the above mentioned systems.

#### 2.2.1. Human as a Threat Object

The driver of a modern car receives information (and help) from various systems: routing, ITS, ADAS. On the one hand, all these systems are designed to make the life of the driver easier and they really can help to solve a wide range of problems the driver routinely faces (e.g., it is hard to imagine a trip without help from a navigation system). However, on the other hand, the greater the veracity of the information sources used by the driver is, the more the driver relies on these sources, the more susceptible the driver becomes to possible malfunctioning of these sources and errors of interaction with them.

There are three potential problems with the information flows directed to the driver, which may affect the user experience of the driver and in some situations even his/her safety.

- Veracity of the information delivered to the driver. Presenting the driver outdated or simply false information about the current state of the infrastructure may cause the driver to make suboptimal decisions. Errors in ADAS may be even dangerous. For example, if left turn assistance system, which monitors the traffic and helps the driver to estimate if the time gap between cars is acceptable, makes an error and classifies a small gap as acceptable, the driver is put into a dangerous situation.
- Timing of information presentation. There is a chance that information presented to a driver attracts his/her attention, when it is critically needed for performing some driving operations.
- Standardization of the interfaces. An important the trend in analyzing a human as a consumer of information provided by vehicle produces a rapid change (and the lack of standardization) of the interfaces caused by new “smart” features of the modern vehicles. As [19] points out, until the beginning of the 21st century the composition of the physical buttons and mechanical gauge were more or less the same for any brand, while DAS, infotainment and navigation systems add a new layer of complexity and interactivity and dramatically change cognitive models. These changes require new standardized interface solutions, especially today, where young urban inhabitant is moving away from car ownership towards the “pay as you go” paradigm [19].

#### 2.2.2. Human as Threat Source

The cognitive state of the driver is one of the key causes of traffic accidents [20]. The authors of [21] present a review of “psychophysiological measures that can be utilized to assess cognitive states in real-world driving environments”. They claim that without psychophysiological measures it would not be possible to evaluate the cognitive state of the driver. The most widely used psychophysiological indices “include: electroencephalography and event-related potentials, optical imaging, heart rate and heart rate variability, blood pressure, skin conductance, electromyography, thermal imaging, and pupillometry”. Some conclusions are made on the efficiency of usage of different indices.

The authors of [22] claim that the best driver performance is achieved at a certain level of stress. If the stress is below that level the driver may experience, for example, drowsiness, and the higher level of stress can be related to distractions or aggressiveness. They also classify the possible causes of the stress level change into: the driver’s condition itself (e.g., lack of sleep or impatience), road and traffic conditions (e.g., congestions or monotonic roads), vehicle conditions (level of noise, malfunctions), and external disturbances (e.g., passengers, gadgets, in-vehicle smart systems, and weather conditions) [22]. All these factors can affect the driver’s level of stress.

Indirectly, a classification of vehicle-related threats can be derived from [23], which studies situation awareness in connection with connected vehicles and Internet of Transportation Things [24]. Since the road accidents can be considered as a possible threat, the factors leading to these should be also considered as possible vulnerabilities. The authors present a semantic network of these factors classified based on their relation to the environment, vehicle, or driver. For example, the following classes of factors are identified for the driver: state (e.g., drowsiness, distraction) and proficiency (e.g., skill level). The vulnerability related to the lack of required skill level is very specific (it cannot be directly used for attacks), however, it has to be taken into account. This is tightly related to the driving behavior and possible threats related to the driver’s age (e.g., [25]) and driving style (e.g., [26]). The psychological aspects of driving from the threat point of view is also a subject of multiple research efforts. Yet in 1984, research was carried out [27] aimed at the analysis of driver behavior including comparison of the threat avoidance by experienced and learner drivers. The author identified several threat avoidance behavior patterns that may lead to different probabilities of consequential accidents.

A driver can be associated in another potential source of threats. Many modern smart systems rely on something that is called participatory sensing. In these systems, “human sensor data” are processed and made available to other users/participants of the system [18]. In this context, it is important to distinguish a human who generates data from a human who is carrying “ambient sensors” to measure external parameters (e.g., air quality with a smartphone). The paper in [28] provides a number of examples of each kinds of sensors. In the first case, the data provided by a human can be purposely erroneous, in the second, a human can try to distort the data collected by sensors. In both cases, it can hamper the efficiency of the system relying on the participatory sensing.

Authors of paper [29] consider driver classification with different Android smartphone sensors. They consider accelerometer, linear acceleration, magnetometer, and gyroscope and investigate which sensor and method are of the highest performance. The classification task is done with different machine learning algorithms like Artificial Neural Networks, Support Vector Machines, Random Forest, and Bayesian Network. The results show that Random Forest performs best followed by artificial neural networks. While, Yang et al. [30] discuss the driver attention detection using eye gaze tracking system. The authors study relationships between the driver’s facial features from first camera, and the eye gaze taken from the second one. The authors used OpenFace analysis tool extracts features of the driver’s head and gaze. The proposed system was tested in three different non-driving activity scenarios, including reading a book, watching a movie on a tablet and playing on a phone. The authors of the paper [31] detected driver’s drowsiness state. The authors detected the following parameters: Head and eye-lid movements (e.g., PERCLOS, blink duration), heart rate and variability, as well as recorded driving behavior, such as vehicle speed, steering wheel angle, position on the lane. The authors proposed AI-based models to detect the drowsiness level every minute and to predict the time to reach a certain level of drowsiness. Experiments provide data for these models with participants who drove a car simulator under certain conditions.

### 2.3. Research Questions And Method

Based on the analysis of recent research papers as well as on our experience in the topic of DAS development [32,33,34], we have identified research topics in the area of DAS that at the moment require attention from the scientific community. As a result, the following research questions have been identified for the paper:

- RQ1: What information flow is supported in Driver-Environment-DAS infrastructure?
- RQ2: Which classes of threats in Driver-Environment-DAS systems are related to safety in vehicle cabin?
- RQ3: Which threats can be detected by smartphone sensors?

We are approaching these questions through the following method. Based on the state of the art literature and DAS models analysis, we generalize the main threats in vehicle cabin and classify them from the following points of view: Information flows in the cabin, communicating entities, and detectability via smartphone sensors. Since we could not find any sources analyzing the threat detectability via smartphone sensors, it can be considered as a novel contribution. Then, we validate our findings via a developed prototype.

## 3. Classification of Potential Threats in Vehicle Cabin

We have identified possible threats in the vehicle cabin based on the related work analysis. To present them in a systematic way, it is useful to consider them in the context of entities and information flows in the vehicle cabin. Any of these threats can be associated with either an entity (e.g., state properties of it), or with some of the information flows. We present the influence diagram, showing the entities, the information flows, and map the identified threats to the elements of this influence diagram. Also, we present vulnerability classification in the vehicle cabin. After that, we propose threats classification and highlight which of them can be detected by smartphone sensors.

### 3.1. Driver-Environment-DAS Information Flows

**Figure 2:**
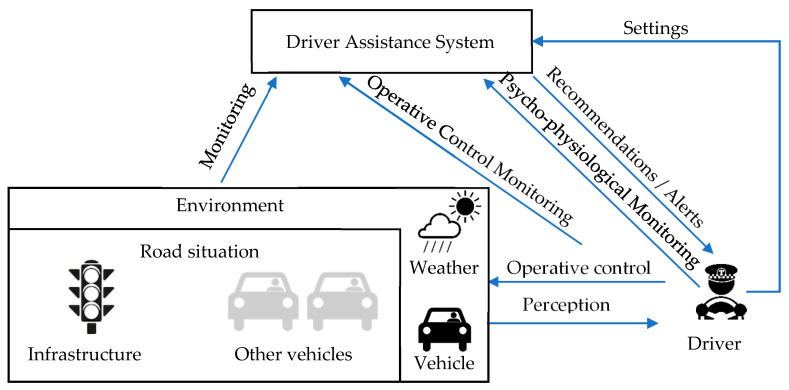
Influence diagram as a framework for threat analysis.

Figure 2 presents the influence diagram showing relevant entities and their interdependencies. Main entities are a driver, an environment, and a driver assistance system. We differentiate a driver assistance systems from advanced driver assistance systems, which is an integrated system installed to the vehicles on manufactures. In the considered case, the driver assistance system implements the functions of driver monitoring. The environment is a complex entity, which includes a set of other entities influencing the safety and security of the driver, namely: vehicle, weather, and road situation. Vehicles in this context represent all the factors attributed to the vehicle used by a driver: Ergonomics of the controls, technical condition, and even passengers who share the trip with the driver. A road situation includes all the entities introduced by the transportation system: infrastructure (including road, semaphores, traffic signs, and road markings) and other vehicles in the neighborhood of the driver’s vehicle. Finally, weather represents factors of the environment not related to the transportation system, but affecting the safety and security (e.g., fog, heavy rain). For example, most of the threats resulted from abnormal driver state should be attributed to the driver in this diagram. Threats resulted from bad road conditions to the infrastructure, and from traffic situation to other vehicles.

Interdependence structure consists of two levels. The first level is the driver, the environment, and the interactions reflecting the fact that the driver continuously monitors the environment and makes operative driving decisions. This circuit exists as long as people drive vehicles. On the other hand, recent developments in the area of driver support have added a new level to this picture. Namely, sometimes, there is some entity (a system running either on the in-vehicle hardware or on a smartphone attached to the windshield) that monitors the driver and/or the situation and issues some alerts/recommendations to increase driver’s comfort and safety. The presence of this system transforms the situation in several ways. First, the system can detect some deviations in the driver’s behavior and/or anomalies in the information flows and warn the driver. Second, the system is itself associated with a number of information flows that can be distorted, resulting in potential threats. Finally, the presence of such system can change the behavior of the driver, who can rely on the system and make mistakes in case the system fails unexpectedly.

The logic behind using the diagram in Figure 2 as a source for threat identification and structuring is explained. First, the ‘lower level’ cycle: A driver percepts the road situation, processes it according to the set of driving patterns and habits, arrives to some operative decisions and implements them with a help of some typical human machine interface (HMI) in the cabin (driving wheel, pedals etc.). Each step of this cycle has its own associated threats: Perception can be influenced by some distractions or bad visibility, situation processing may be inadequate due to poor training and low driving experience, finally, operative control implementation may fail because of vehicle’s technical conditions and limitations. Not all of these threats are easily avoidable or even observable, but, some are. The ‘upper level’ cycle involves DAS, which monitors road situation, operative control decisions taken by the driver, and the driver’s state to alert the driver in case of dangerous behavior. In particular, by monitoring the driver’s state, DAS checks whether the driver is alert and may percept the road situation. By monitoring the road situation and the operative decisions of the driver, it may to some extent assess the driving habits and issue recommendations for improving them. However, the presence of the DAS brings four new information arrows and one entity and each of them have some threats associated. For example, failure of driver state monitoring information flow or algorithm may result in inadequate recommendations distracting and/or annoying the driver. Errors in road situation monitoring may again lead to inadequate or even dangerous recommendations. Besides, some DAS also can be configured (a driver can set alert thresholds, fine-tune the system’s behavior) or even enabled/disabled by the driver (the Settings arrow in Figure 2 captures all these possibilities). It can also pose some threats to the driver and/or other traffic participants.

### 3.2. Vulnerability Classification in Driver–Environment–DAS

Based on the interacting entities of the Driver-Environment-DAS system and information flows between them we identified potential threats to safety, following the idea that each threat can be associated with an entity, or with an information flow (Table 1). We have generalized the main threats in the vehicle cabin based on the literature review and analysis of existing DAS models. For each entity or information flow identified in Section 3.1, the table contains a brief description of the entity/flow, and possible threats. Threats are identified based on an observation that a threat can be caused, either by the state of some entity, or by corruption of an information flow. However, flows and entities cannot be analyzed totally independently, as flow contents is determined by its source entity. That is, establishing why, in some cases, entity-associated threats have flow-associated counterparts. For example, abnormal driver state is an entity-associated threat, but it results in inadequate driving decisions that are the contents of the operative control information flow, which is also a flow-associated threat.

### 3.3. Threats Classification

This section aims at threat classification with the purpose of defining which classes of threats can be detected via usage of smartphone. On this basis, the classification from [17] was used. In order to define sources and aims of threats, the vulnerabilities, attacks and threats that can take place in the Driver-Environment-DAS system were considered. On the other hand, the classification of threats based on their nature (namely, cyber, cyber-physical, physical, psycho-physiological, and cyber-psycho-physiological) was carried out (Table 2).

The threat sources column includes information on the evaluated possibility of threat detection via smartphone sensors. Unlike the attacker, the attack target (e.g., confidentiality, integrity, availability) does not affect the possibility of threat detection by smartphone sensors, it is not considered in this section. 

We could not find any works that would address cyber and cyber-physical threats via smartphones. Hence, we claim that these threats cannot be detected via smartphone.

Physical threats from passengers or from outside of the vehicle can be partly detected via the smartphone. For example, the acceleration sensors can measure sharp acceleration or stopping related to, for example, an accident, however, this can be only be done after the incident (e.g., [46]). On the other hand, it is possible to identify, for example, presence of the dirt on the windshield via the smartphone camera. We could not find any research works in this area, however, we do not exclude this possibility.

Detection of misuse by the driver of some vehicle HMIs by smartphone is possible and have been researched in multiple efforts. For example, detection of the proper usage of seatbelts [40], keeping the driver’s hands on the steering wheel [47] and others.

Unpredictable behavior of DAS can also be a threat (for example, emergency braking in front of non-existing obstacle may cause a collision if the driver of the vehicle behind does not expect such braking). Obviously, a smartphone cannot detect such threats, however, certain rules can be developed (e.g., sharp acceleration by driver following sharp braking by DAS) that would help to generate reports for DAS developers to improve their systems in the future.

Detection of psycho-physiological threats with the help of smartphone is currently devoted more attention than to other classes of threats. For example, smartphones can be successfully used for identification of extra noise (e.g., [48]) that can be distracting for the driver (e.g., talking to passengers). In the similar way, the behavior of DAS can be evaluated for future reports. For example, when the system provides warnings too often (in this case the driver can become nervous, will not pay attention to signals from DAS, and can overlook a dangerous situation [49,50]) or if the system works fine and the driver relies on it too much, and then the system fails (e.g., [51]). Such reports can be generated by a smartphone via an analysis of the frequency of DAS signals together with the driver’s behavior.

The abnormal driver state and behavior can also be partially detected via smartphone sensors. (drowsiness, distraction, etc.) via AI-based analysis of images taken by the smartphone camera [32]. The following section describes this in detail.

As an analogy to distinguishing between physical and cyber-physical attacks, we distinguish between psycho-physiological and cyber-psycho-physiological influence. Whereas in the former the driver’s stress level is influenced in a physical way: Through physical interaction (e.g., pushing the driver), visual (e.g., visual image that makes the driver angry) or audio (e.g., talking to the driver to make him/her angry); the latter changes the driver’s stress level through internet, cellular network or radio (e.g., delivering information that make the driver angry). The simplest example is irritating spam phone calls or messages. We have not been able to find studies related to driver safety and currently cannot suggest any mechanisms to detect such threats using mobile phone sensors. Only the consequence related to changing driver’s mood could potentially be detected, but not the threat itself.

## 4. Case Study

In the previous section we have defined threats and classified them. The role of the case study is to demonstrate implementability of the identified threats detection via smartphone sensors within feasible time frames.

Based on our previous research work [32], we identified the main use cases for threats identification using smartphone sensors:

- drowsiness (pre-sleeping condition, the driver concentration to the road is decreasing);
- distraction (the driver does not concentrate to the road: the head is turned left/right/top/down);
- unfastened belt (the belt is not fastened what causes a threat to the driver’s life);
- eating (distraction from the road caused by eating something);
- drinking (distraction from the road caused by drinking something);
- smartphone use (distraction from the road caused by talking or messaging via smartphone).

**Figure 3:**
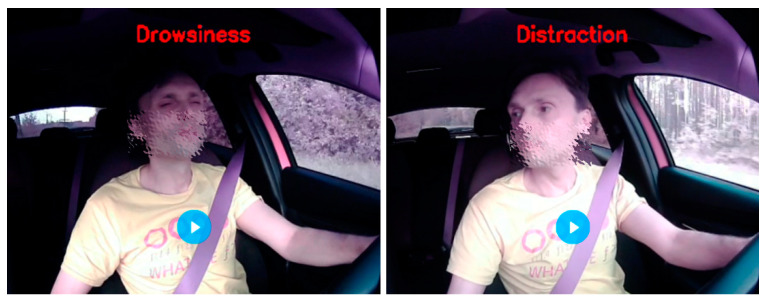
Drowsiness and distraction use cases.

**Figure 4:**
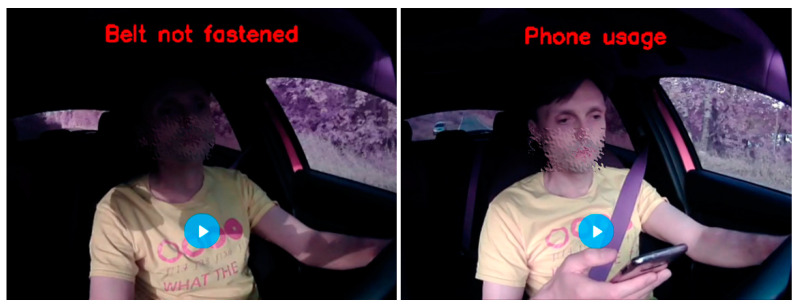
Unfastened belt and smartphone usage use-cases.

We developed a software framework to support these use cases. The driver can record a video in vehicle cabin and then check it using the framework. The framework utilizes the Faceboxes neural network model [52] to detect the driver’s face and the Dlib framework to detect facial landmarks. Based on these landmarks, the drowsiness and distraction use cases are detected (Figure 3). For the unfastened belt, eating, drinking, smoking, and smartphone use cases (Figure 4) we developed a neural network model based on the YOLOv3 framework and a dataset that currently includes more than 3000 images of drivers in vehicle cabin. We use Java programming language to develop the cross platform prototype for the smartphone-based video analysis.

We conducted experiments (see Table 3) and estimated accuracy of the developed framework (described in details in [32,40]) based on in-vehicle experiments. The experiments were conducted for one driver during several hours of driving. The driver performed a sequence of actions, defined by an operator. After that, the results of video recognition obtained using the proposed framework were compared with the operator data.

## 5. Discussion

In this section, we summarize the answers to the research questions specified in the introduction and analyzed in the paper. We recite the research questions one by one and provide answers based on the carried out analysis.

*RQ1: What information flow is supported in Driver-Environment-DAS infrastructure*?

We have identified major entities related to the process of driving a vehicle and major information flows between these entities. The entities we consider are: driver, environment (including the vehicle being controlled by the driver, road situation), and DAS. There are following flows between these entities: perception, operative control, monitoring (of the environment, of the driver), and possibly configuration (of the DAS by the driver). Based on the fact, that any threat can be associated with either an entity or an information flow, this analysis allows to systematically enumerate threats relevant to the process of driving a vehicle and further identify the threats that can be detected and assuaged by a smartphone.

In particular, the analysis of information flows (and the respective diagram, shown in Figure 2 helps to identify some threats introduced by DAS. On the one hand, DAS helps to avoid possible accidents by monitoring the driver and the environment, on the other hand it introduces a number of entities and flows, each of which has its own threats associated (e.g., inadequate DAS models, alerts in an inappropriate moments etc.). It also should be noted, that the approach to detect threats via information flow analysis can be extended to account for new entities, either on the same level of abstraction (e.g., car infotainment system), or on more fine-grained levels (e.g., decomposing DAS into typical subsystems and taking into consideration information flows between them).

*RQ2: Which classes of threats in Driver-Environment-DAS systems are related to safety in vehicle cabin*?

Based on the identified entities and information flows, as well as on the literature analysis, we have identified a number of safety-related vulnerabilities. Each of these vulnerabilities is connected either to vulnerabilities of an entity (its state), or to the information flow (its corruption). We distinguish the following threat classes based on vulnerable entities and flows.

- Driver assistance systems;
- Driver;
- Monitoring;
- Recommendations/Alerts;
- Operative Control;
- Perception.

We also found it useful to classify the identified vulnerabilities and associated threats to five classes, depending on the kind of the threat target, as well as on what channels and mechanisms are used to fulfil the attack: cyber, cyber-physical, physical, psycho-physiological, and cyber-psycho-physiological. Below, we identify detectable by smartphone threats based on this classification.

*RQ3: Which threats can be detected by smartphone sensors*?

It was found that smartphones cannot be used to identify any threats related to cyber communication channels. Therefore, we have not found any evidence that cyber or cyber-physical threats (not only related to safety, but also to privacy) can be detected by smartphone sensors. The same is the truth for cyber-psycho-physiological threats that we have proposed as a new class of threats. 

However, the availability of various sensors is a significant advantage of using smartphones for detecting threats related to physical world. For example, physical threats from outside of the vehicle can potentially be partly detected (for example, the presence of dirt on the windshield via the smartphone camera). However, we could not find any research works in this area. Detection of misuse by the driver of some vehicle HMIs by smartphone is possible and have been researched in multiple efforts (proper usage of seatbelts, keeping the driver’s hands on the steering wheel, etc.). The unpredictable behavior of DAS cannot be timely detected by smartphone, but a system can be developed that would produce reports with potential errors made by DAS.

Detection of psycho-physiological threats by smartphone at the moment is one of the most popular research topics. Various threats can be detected from abnormal driver state to annoying DAS signals or extra noise.

We have also identified a new class of threats, namely cyber-psycho-physiological threats related to influencing the driver’s stress level via telecommunication channels (internet, cellular network, or radio). Their examples include irritating spam phone calls, messages or radio translations. No studies related to the effect of this threat to the driving safety have been found so we conclude that at the moment they cannot be detected by smartphone. However, it can be noted that consequences related to changing driver’s mood could potentially be detected by smartphones.

Of course, we understand that usage of smartphones is not an optimal solution for threats identification. However, the identified approaches can be adopted in the in-vehicle embedded systems for increasing the car safety level.

## 6. Summary and Future Work

The paper presents research results in the area of potential threats detection based on smartphone sensors in the vehicle cabin. We discuss the modern intelligent transportation systems and conclude that together with benefits such systems bring potential vulnerabilities. We have identified possible threats in the vehicle cabin. We presented the influence diagram, showing the entities and the information flows, and mapped the identified threats to the elements of this influence diagram. Then, we presented vulnerability classification in vehicle cabin. After that, we proposed threats classification and highlighted which of them can be detected by smartphone sensors. We classified threats as cyber-physical, physical, psycho-physiological, and cyber-psycho-physiological. As the result, we have introduced a new class of cyber-psycho-physiological threats that have not been presented in the literature before. Threat classes were illustrated with examples.

In the future, we plan to conduct research on algorithms that would evaluate correlation between driver and DAS actions. This, for example, would make it possible to evaluate the driver reaction time [42], which could indirectly indicate the driver’s state, false DAS alarms (when no actions is undertaken after the alarm and no consequences occur), false DAS evasive actions (e.g., braking or steering by DAS immediately compensated by the driver’s actions without consequences), and others. Another direction of the future research is to study the benefits of smartphone connection to CAN bus via On-board diagnostics (ODB) Bluetooth dongles. Such connection provides additional possibilities to analyze additional parameters for vulnerability detection in vehicle cabin.

## Figures and Tables

**Table 1:** Vulnerability classification for driver monitoring systems.

Vulnerable Entity/Flow	Description	Threat
**Entity-Associated**		
Driver assistance system	Provides high-level (local driving restrictions, congestion and routing) and low-level (maneuver, lane change, dangerous state detection) assistance and monitoring. May build a personalized driver model to match driver’s style.	- Inadequate DAS models causing the incorrect system behavior. - Inadequate DAS settings causing recommendations inconsistent with the driving habits. - Errors in state estimation caused by inadequate driver model.
Driver	Processes perceived information with a set of driving habits.	- Lack of training, suboptimal operative decisions, bad driving habits. - Abnormal driver state (drunk, drowsy, etc.) may hamper the perception of the situation and driver may miss some important changes in the road situation to react reasonably. - Different types of distraction and inattentive driving (mobile phone and smartphone usage, smoking, etc.). - Other safety requirements violation (seat belt unfastened, etc.).
**Information flow-associated**		
Monitoring	The process of observing the state of some entity and usually matching it with the desirable (or acceptable) state. It can be divided into environment monitoring, operative control monitoring, and psychophysiological monitoring.	Cheating state estimation for disabling alerts by smartphone usage, detection of unfastened seatbelt, etc.; sensors tampering, etc.
Recommendations/Alerts	Signals issued to the driver by DAS to draw the driver’s attention to some important changes in the situation or to inform about reasonable actions. Can be high-level (recommended speed, route) and low-level (maneuver suggestions, dangerous state alerts).	- Recommendations inconsistent with the driving habits or erroneous (recommendation to make a maneuver when it is dangerous) may result in suboptimal routing decisions or traffic rules violation (high-level recommendations), a dangerous situation, or even an accident (low-level recommendations). - Recommendations provided in an inappropriate moment, distracting or stressing the driver may result in lowering driver’s attention, which, in turn, may result in dangerous situation or car accident, and also in lowering the user experience causing turning the system off.
Operative Control	A driver implements his/her driving decisions (maneuvering, speed control etc.) with a help of some typical HMI in the cabin (driving wheel, pedals etc.).	- Inadequate driving decisions (caused by bad driving habits or abnormal state) may result in the increase of accident chances. - Malfunction of the cabin HMI (including, intercepting the signals from the HMI to prevent the vehicle doing something requested by the user).
Perception	A driver percepts the road situation.	- Missing important changes in situation (e.g., due to abnormal state, visual and audial obstacles, compromised HMIs reporting incorrect statuses to attempt driver or passengers to perform certain actions) results in the increase of accident chances.

**Table 2:** Vulnerabilities, attacks, and associated threats detectable by smartphone sensors.

	Vulnerability	Attack	Threat Source
Cyber	Various cyber vulnerabilities (e.g., packet injection, malware injection, etc.) [13,14,15,16,17].	Non-physical interaction with the vehicle, its communication channels, or other systems the vehicle interacts with [13,14].	Environment (passenger or something/somebody from outside) **Cannot be detected via smartphone** Driver (unintentionally or intentionally) **Cannot be detected via smartphone**
Cyber-Physical	Vulnerability to sensory channel attack (manipulating the physical environment to deceive vehicle’s sensors) [16,35,36,37], hardware tampering to infect with malware [16] or installing additional hardware (e.g., for eavesdropping [17]), insecure CAN bus [38], and other.	Manipulating the physical environment to deceive vehicle’s sensors [16,35], replay attacks (retransmitting previously captured legitimate commands [39].	Environment (passenger or something/somebody from outside) **Cannot be detected via smartphone** Driver (unintentionally or intentionally) **Cannot be detected via smartphone**
Physical	Vulnerability to hardware tampering [16,17], hardware failure [16].	Physical damaging (including accidents) or natural degradation of vehicle’s components) [16].	Environment (passenger or something/somebody from outside) **Can partially be detected via smartphone (e.g., dirt on the windshield, etc.)** Driver (unintentionally or intentionally) **Can partially be detected via smartphone (e.g., hands on the steering wheel, seatbelt usage, etc.)** [40] DAS (unpredictable behavior) **Cannot be detected via smartphone in real time, but reports can be generated**
Psycho-physiological	Change of stress level [22] (including drowsiness, irritation, aggressiveness, distraction, etc. [21,23,41]), driver’s physical condition change, driving skills level [23], driver’s age [25], driving style [26], other driver characteristics [27,42,43], other vulnerabilities (e.g., solar glare vulnerability [35]).	Unwanted interaction with the driver (sound, light, physical interaction) [22] or absence of the expected interaction [44].	Environment (passenger or something/somebody from outside) **Can partially be detected via smartphone (e.g., noise level measurement)** DAS (annoying, false alarms, failures in detecting threats). **Can partially be detected via smartphone (e.g., frequency of DAS alarms)** The driver (drowsiness, heart attack, external locus of control etc.) **Can partially be detected via smartphone (e.g., frequency of DAS alarms) ** [22,32,45]
Cyber-psycho-physiological	Change of stress level [22] (including drowsiness, irritation, aggressiveness, distraction, etc. [21,23,41]).	Unwanted interaction with the driver through communication channels (e.g., phone calls, messaging).	Environment (passenger or something/somebody from outside) **Can partially be detected via smartphone (e.g., noise level measurement)**

**Table 3:** Developed framework evaluation.

#	Use-case	Recall	Precision
1	Drowsiness	69%	95%
2	Distraction	95%	98%
3	Belt Unfastens	70%	100%
4	Eating/Drinking	45%	80%
5	Smartphone Usage	87%	92%
